# Positive selection and climatic effects on *MHC* class II gene diversity in hares (*Lepus capensis*) from a steep ecological gradient

**DOI:** 10.1038/s41598-018-29657-3

**Published:** 2018-07-31

**Authors:** Asma Awadi, Hichem Ben Slimen, Steve Smith, Felix Knauer, Mohamed Makni, Franz Suchentrunk

**Affiliations:** 1 Unité de Recherche Génomique des Insectes Ravageurs des Cultures d’Intérêt Agronomique, Faculty of Sciences of Tunis, University of Tunis El Manar, 2092 Tunis, Tunisia; 2 Institut Supérieur de Biotechnologie de Béja, University of Jendouba, Avenue Habib Bourguiba Béja 9000, BP. 382 Béja, Tunisia; 3 Research Institute of Wildlife Ecology, University of Veterinary Medicine Vienna, Savoyenstrasse 1, 1160 Vienna, Austria

## Abstract

In natural populations, allelic diversity of the major histocompatibility complex (MHC) is commonly interpreted as resulting from positive selection in varying spatiotemporal pathogenic landscapes. Composite pathogenic landscape data are, however, rarely available. We studied the spatial distribution of allelic diversity at two MHC class II loci (*DQA, DQB*) in hares, *Lepus capensis*, along a steep ecological gradient in North Africa and tested the role of climatic parameters for the spatial distribution of *DQA* and *DQB* proteins. Climatic parameters were considered to reflect to some extent pathogenic landscape variation. We investigated historical and contemporary forces that have shaped the variability at both genes, and tested for differential selective pressure across the ecological gradient by comparing allelic variation at MHC and neutral loci. We found positive selection on both MHC loci and significantly decreasing diversity from North to South Tunisia. Our multinomial linear models revealed significant effects of geographical positions that were correlated with mean annual temperature and precipitation on the occurrence of protein variants, but no effects of co-occurring *DQA* or *DQB* proteins, respectively. Diversifying selection, recombination, adaptation to local pathogenic landscapes (supposedly reflected by climate parameters) and neutral demographic processes have shaped the observed MHC diversity and differentiation patterns.

## Introduction

Genetic studies of natural populations based on presumably neutral markers such as mitochondrial d-loop DNA (mtDNA), microsatellites or SNPs are important to infer phylogeny, population history and gene flow^1,2^. However, in some cases the time span between the separation of populations might be too short or gene flow might be too strong to leave a signal of differentiation at neutral loci^3^. In such cases, differences between populations can only be detectable at functional genes under selection^3^. Moreover, studying molecular polymorphism at loci under natural selection will help to understand the genetics of adaptive processes and to increase our knowledge on the importance of adaptive genetic variability in free ranging animal populations^1^. Local adaptation at functional markers has mainly been addressed in populations structured at neutral markers and rarely in the case of seemingly panmictic populations^4^. A study of Tunisian hare populations indicated a strong population structure at mitochondrial OXPHOS genes, which are under positive selection at several codons, despite a high gene flow at neutral markers^5^. These findings are consistent with local adaptation according to climate variation^5^.

Adaptation to local or regional pathogenic landscapes is often addressed by analyses of major histocompatibility genes (MHC) which code for glycoproteins that recognize and bind antigens in order to present them to CD4^+^ and CD8^+^T-lymphocytes to initiate the adaptive immune response against pathogens. MHC class I and class II loci, the two main types of MHC loci involved in the adaptive immune system, have been shown to be highly polymorphic in a wide range of species^6–9^ and their adaptive significance in vertebrates has long been recognized^10,11^. Allelic diversity at these loci can be associated with variation across environmental and geographical scales as demonstrated from several studies^12,13^. The role of environmental characteristics in shaping selective pressures on MHC genes is suggested to be mediated by combined effects including recent and ancient demography as well as behavioural and ecological differences in pathogens exposure^14–16^. Moreover, host-pathogen interactions have been suggested to be affected by temperature, nutrient availability and environmental stress^17^. Whereas increases of ambient temperature can reinforce selection on immune genes, thereby accelerating host adaptation^18,19^, variation in nutrient levels can force physiological modification that could either impair or promote local adaptation of the host^17^.

Among all MHC molecules, class II genes have received the most attention, due to their role in studying mechanisms and significance of molecular adaptation in vertebrates^20^ and host parasite co-evolution^2^. MHCII molecules are heterodimers formed by an α and a β chain encoded by A and B genes, respectively^21,22^. These chains are characterized by several typical structures, such as N-linked glycosylation sites, connecting peptides, transmembrane regions, and cytoplasmic domains, as well as the α1/α2 domain and the β1/β2 domain, respectively^23^. The α1 and β1 domains constitute the peptide-binding region (PBR), involved with the recognition and binding to the antigens^14,24,25^. These domains are highly polymorphic and are encoded by the second exon of A and B MHCII genes. Given their role in presenting antigenic determinant peptides to the cell surface, these loci are typically expected to be subject to strong positive selection, since their greater allelic diversity should be associated with a response to a wider range of pathogens^16^. Genetic diversity in the MHC is also generated through several mechanisms, including point mutation, recombination, gene conversion, sexual selection and maternal–foetal interactions^25–28^. The variation generated by these processes is thought to be maintained in individuals and populations by a combination of balancing and diversifying selection, driven primarily by variation in selection pressure from pathogens and parasites across space and time^3,28^. Balancing selection encompasses a number of different evolutionary mechanisms that result in a large number of alleles being maintained in populations for longer than would be expected relative to neutral genetic variation^24^. Such balancing selection is generally explained by three mechanisms a) over-dominant selection or heterozygote advantage where heterozygous individuals have higher fitness than both corresponding homozygotes; b) frequency dependent selection or rare allele advantage where individuals with rare genotypes have higher fitness^29,30^; c) fluctuation in selection pressure where MHC polymorphism is maintained by changing pathogen composition through time and space. Conversely, under diversifying selection at MHC loci, the spread of an advantageous allele (positive selection) would be expected to lead to a loss of genetic variation. Similarly, selection against disadvantageous alleles (negative or purifying selection) would also be expected to reduce diversity^31^. Among the different suggested mechanisms of balancing selection, fluctuation in selection pressure have been reported for class II MHC genes among populations of the Asian cygomolgus macaque (*Macaca fascicularis*)^12^, and the Atlantic salmon^32^, among other studies. Patterns of MHC selection can be inferred by comparing MHC genetic differentiation to differentiation at presumably neutral markers^28^. Neutral loci reflect demographic factors such as migration and drift, whereas loci under selection usually show higher differentiation under different local selection regimes or lower differentiation under balancing selection^28,33^ or under negative/purifying selection^33^. Higher differentiation at MHC genes could therefore reflect local adaptation to different parasite faunas prevailing in the respective populations.

In Tunisia, hares (*Lepus capensis*) are distributed throughout the whole country in diverse environments and over short geographic distances, reaching from the Mediterranean climate in the north to the pronounced arid desert climate (Sahara) in the south. This situation provides a good precondition to study natural selection and adaptation on functional genes^5^. The likely differences in differential parasite pressure in these habitats could lead to different selection pressure at MHC loci among the local populations. Such selective pressure would shape diversity and differentiation profiles in interaction with neutral demographic/stochastic factors. In this study, we examined the MHC class II polymorphism for *DQA* and *DQB* genes in hares from Tunisia along a steep ecological gradient. We first investigated the variation of genetic diversity in the different studied populations distributed in heterogeneous ecoregions from North to South. If diversifying selection is the main evolutionary force acting on MHC genes, we expected significant genetic differentiation despite observed high gene flow in neutral markers. Second, we used different approaches to evaluate the effect of selective pressure in each codon of the obtained sequences. Moreover, we compared the levels of allelic diversity of the considered hare populations with those obtained for a set of neutral loci. We expect that MHC diversity varies significantly between the regional populations under study given that spatial changes in parasite communities and dynamics with environmental conditions might alter the selection pressures they exert on their host populations^17^. However, neutral demographic processes (i.e., gene flow, genetic drift…) can also have significant effects on the genetic diversity of natural populations^4,34^.

Given the steep ecological gradient along the relatively short geographical distance from where our samples were drawn and the absence of any obvious migration barrier across our study area, significant variation of MHC allele frequencies across the study area should reflect adaptation to local environments. Thus, we hypothesize that climatic factors associated with the geographical origin of the samples will lead to spatial variation in the frequencies of the most common alleles. Moreover, MHC alleles at different loci are expected to co-evolve. Thus, we further expect a significant level of linkage between the respective co-occurring allele or genotype at the second MHC locus studied on the occurrence of an MHC allele under consideration. Such allele effects of co-occurring MHC loci might have led to an “optimal” combination of alleles at the two loci studied independently of spatial or climatic effects.

## Results

### Allelic diversity and genetic differentiation

**Figure 1:**
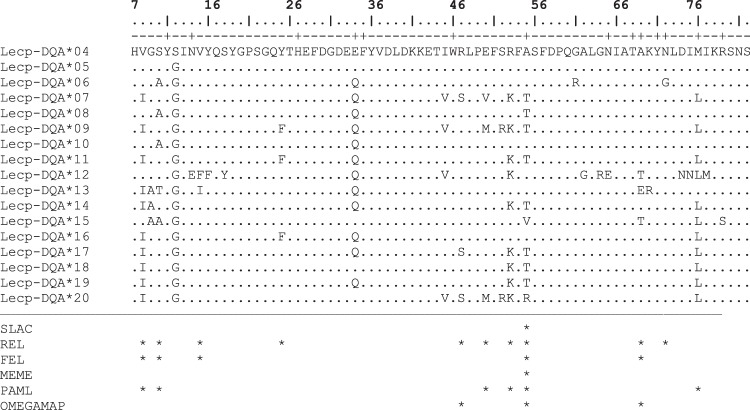
Amino acid alignment of DQA exon 2. Antigen-binding sites (ABS) are indicated above with the sign “+”. Positively selected codons identified by the different methods are indicated with asterisks. Numbering of amino acid residues is according to Bondinas *et al*.^89^.

**Figure 2:**
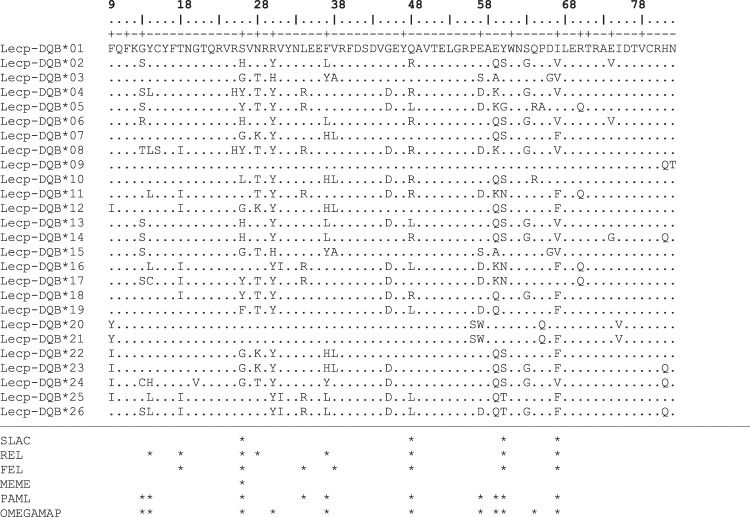
Amino acid alignment of *DQB* exon 2. Antigen-binding sites (ABS) are indicated above with the sign “+”. Positively selected codons identified by the different methods are indicated with asterisks. Numbering of amino acid residues is according to Bondinas *et al*.^35^.

We identified 17 *DQA* alleles (accession numbers MH346126-MH346142) and 26 *DQB* alleles (accession numbers MH346143- MH346168) in 142 hares (*L. capensis*) from Tunisia with a total length of 228 and 222 bp, respectively. Among them, only three DQA alleles (Lecp-DQA*04, Lecp-DQA*05, Lecp-DQA*06 corresponding to Leeu-DQA*02, Leeu-DQA*03, Leeu-DQA*09 in *L. europaeus*, respectively) were previously described in *L. europaeus*. No insertions, deletions, or stop codons were detected in either *DQA* or *DQB* sequences. The nucleotide sequence of each allele in both loci translated into a different amino-acid sequence (Figs 1 and 2). Allele frequencies and basic diversity parameters for the two MHC class II exon 2 loci in the different populations as well as the overall values are displayed in Table 1. Hares were grouped into three populations according to climatic, geographic and phenotypic data (see methods section). These populations are northern Tunisia (NT), central Tunisia (CT) and southern Tunisia (ST).

**Table 1:** Allele frequencies of two MHC class II loci for the three Tunisian hare populations.

DQA Allele	NT	CT	ST	DQB Allele	NT	CT	ST
***Lecp-DQA*** *04		0.0075		***Lecp-DQB*** *01	0.0500	0.3219	0.0286
***Lecp-DQA*** *05	0.0484	0.0149		***Lecp-DQB*** *02	0.0500	0.1027	0.0143
***Lecp-DQA*** *06	0.0161	0.0075		***Lecp-DQB*** *03		0.0411	0.5000
***Lecp-DQA*** *07	0.3548	0.1493	0.0690	***Lecp-DQB*** *04	0.0167	0.0479	
***Lecp-DQA*** *08	0.2258	0.5746	0.1034	***Lecp-DQB*** *05		0.0068	0.2143
***Lecp-DQA*** *09	0.0323	0.0597	0.0345	***Lecp-DQB*** *06	0.0333	0.1233	
***Lecp-DQA*** *10	0.0161	0.0224	0.1379	***Lecp-DQB*** *07	0.0333	0.1507	
***Lecp-DQA*** *11	0.0806	0.0970	0.3793	***Lecp-DQB*** *08		0.0411	0.0714
***Lecp-DQA*** *12	0.0161	0.0149	0.0345	***Lecp-DQB*** *09	0.4667	0.0411	0.0143
***Lecp-DQA*** *13			0.0690	***Lecp-DQB*** *10		0.0068	0.0286
***Lecp-DQA*** *14	0.0161	0.0075		***Lecp-DQB*** *11		0.0137	0.0429
***Lecp-DQA*** *15	0.0161	0.0299		***Lecp-DQB*** *12	0.0167	0.0685	0.0286
***Lecp-DQA*** *16	0.0323	0.0149	0.1034	***Lecp-DQB*** *13		0.0137	
***Lecp-DQA*** *17			0.0690	***Lecp-DQB*** *14	0.0500		
***Lecp-DQA*** *18	0.0161			***Lecp-DQB*** *15	0.0167		
***Lecp-DQA*** *19	0.0968			***Lecp-DQB*** *16	0.0500	0.0068	
***Lecp-DQA*** *20	0.0323			***Lecp-DQB*** *17			0.0143
				***Lecp-DQB*** *18	0.0333		0.0429
				***Lecp-DQB*** *19		0.0137	
				***Lecp-DQB*** *20	0.0667		
				***Lecp-DQB*** *21	0.0167		
				***Lecp-DQB*** *22	0.0167		
				***Lecp-DQB*** *23	0.0167		
				***Lecp-DQB*** *24	0.0167		
				***Lecp-DQB*** *25	0.0333		
				***Lecp-DQB*** *26	0.0167		
**N**	31	67	29	**N**	30	73	35
**Na**	14	12	9	**Na**	18	15	11
**H exp**.	0.8002	0.6323	0.7990	**H exp**.	0.7611	0.8351	0.6922
**H obs**.	0.4839	0.4776	0.5517	**H obs**.	0.5000	0.3425	0.4571
**Rs**	13.603	9.050	9.000	**Rs**	17.731	11.941	10.396

N Sample size, Na number of alleles, Hobs. observed number of heterozygotes, Hexp. expected number of heterozygotes, Rs allelic richness, NT: northern Tunisia, CT: central Tunisia, ST: southern Tunisia.

The numbers of alleles per regional population were 14, 12, and 9 in NT, CT, and ST, respectively, for the DQA locus. For *DQB*, allele numbers were 18, 15, and 11 in NT, CT and ST, respectively (Table 1). In general, in all three populations, few alleles were relatively abundant, e.g. Lecp-DQA*07, Lecp-DQA*08, *Lecp*-DQB*01 and *Lecp*-DQB*09 were highly frequent, whereas most others were detected at very low frequencies.

A comparison of the obtained genotypes revealed alleles specific to each regional population. For *DQA*, seven alleles were shared between the three populations, whereas three alleles (Lecp-DQA*18, Lecp-DQA*19, Lecp-DQA*20) were specific to NT, one allele (Lecp-DQA*04) to CT and two (Lecp-DQA*13, Lecp-DQA*17) to ST. For *DQB*, only four alleles (Lecp-DQB*01, Lecp-DQB*02, Lecp-DQB*09, Lecp-DQB*12) were shared between the three regional populations. Nine alleles were specific to NT, two to CT, and two to ST. Frequencies of these specific alleles ranged between 0.0161 and 0.0968 for *DQA* and between 0.0137 and 0.0500 for *DQB* (Table 1). Finally, allelic richness (Rs), which measures the number of alleles independently of sample size, was significantly (p < 0.0001) higher for the two MHC loci than for the microsatellite loci in NT but not in CT and ST. Moreover, allelic richness varies significantly (p = 0.027) between NT and the two other populations (CT and ST). Significant (F_IS_ for NT = 0.384, p < 0.001; F_IS_ for CT = 0.447, p < 0.001; F_IS_ for ST = 0.338, p < 0.001) deviation from Hardy-Weinberg Equilibrium in all three regional populations indicated that the MHC loci contained significantly fewer heterozygotes than expected. Finally, there was no significant linkage disequilibrium between genotypes at the *DQA* and *DQB* locus as indicated by the Linkage disequilibrium test (R = 0.16; p = 0.22).

Genetic differentiation between regional populations was revealed by pairwise comparisons of Dest and F_ST_ values (Table 2). Population differentiation as determined by mean Dest values across the two loci was high and ranged between 0.46 (NT vs CT) and 0.78 (NT vs ST). For the individual loci, Dest values for the *DQA* locus ranged between 0.29 (NT vs CT) and 0.59 (CT vs ST), whereas those for the *DQB* locus ranged between 0.73 (NT vs CT) and 0.96 (NT vs ST). Similarly, pairwise F_ST_ (Table 2) values revealed significant genetic divergence among regional populations ranging between 0.12 (NT vs CT) and 0.19 (NT vs ST). In contrast, pairwise values for the microsatellite loci were low ranging between 0.013 to 0.030 for F_ST_ values and between 0.043 and 0.112 for pairwise Dest values (Table 2). Finally, a hierarchical population structure (AMOVA) based on analyses of variance of gene frequencies was evident in the genetic differentiation across both MHC loci, with 17.13% of the genetic variance attributable to between regional population differences, 4.79% between sampling localities within regional populations, and 78.09% to within sampling localities (P < 0.001, P < 0.05, and P < 0.001, respectively). These estimations for the microsatellite loci were 0.60 (P > 0.05), 4.58 (P < 0.001) and 94.82 (P < 0.001) for the same parameters, respectively.

**Table 2:** Pairwise *F*_*ST*_ and Dest values among Tunisian hare populations for MHC and microsatellite loci.

	NT	CT	ST
**a. F**_**ST**_ **for MHC (upper values) and microsatellite loci (lower values)**			
NT		0.123*	0.180*
CT	0.013		0.187*
ST	0.030	0.028	
b. Dest for *DQA* (upper values) and *DQB* (lower values) separately			
NT		0.29*	0.54*
CT	0.73*		0.59*
ST	0.96*	0.82*	
**c. Dest for MHC (upper values) and microsatellite (lower values) loci**			
NT		0.46*	0.78*
CT	0.043*		0.69*
ST	0.112*	0.101*	

NT: northern Tunisia, CT: central Tunisia, ST: southern Tunisia. *p < 0.001.

**Figure 3:**
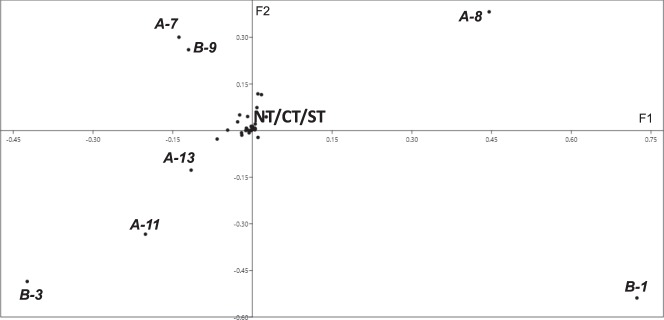
Principal component analysis (PCA) of the allelic diversity in the different sampling regions of hares. Each dot represents one MHC allele; the *DQA* and *DQB* alleles are indicated as A-allele number and B-allele number, respectively.

The first two axes of our PCA (Fig. 3) based on the *DQA* and *DQB* allele matrix explained 31.51% of the variance in our MHC genotypic data (18.19% F1, 13.32% F2). Both axes (F1 and F2) were structured by DQA alleles DQA*7, DQA*8, DQA*11, DQA*13 and DQB alleles DQA*1, DQA*3, DQA*9 (Fig. 3). Among these alleles, only DQA*13 was a rare allele.

Finally, bottleneck signatures were detected in CT (p = 0.0176) and ST (p = 0.0017) populations when using the Two Phase Mutation model (TPM). No tests detected a bottleneck in the northern Tunisian (NT) population. Different Ne estimates were obtained for each population depending on the used method (Table 3). The Heterozygote Excess based method gave an infinitely large estimate of Ne for all populations. The smallest estimates were observed in the ST population (317.9) by the Linkage disequilibrium method.

**Table 3:** Estimates of effective population size (Ne) according to three different methods implemented in Ne estimator.

		Linkage disequilibrium	Heterozygote Excess	Molecular Coancestry
**NT**	Ne	396.8	Infinite	364.0
	95% CI	123.4-Infinite	—	0.4–1827.5
**CT**	Ne	477.2	Infinite	Infinite
	95% CI	274.3–1588.2	—	—
**ST**	Ne	317.9	Infinite	Infinite
	95% CI	109.2-Infinite	—	—

NT: North Tunisia, CT: Central Tunisia, ST: South Tunisia. CI: confidence interval.

### Selection and recombination analysis

Using the three PERMUTE statistics, no recombination was obtained for the *DQA* locus, whereas recombination evidence was obtained by one test (r^2^ = −0.163; p = 0.001) for the *DQB* locus (Table 4). One recombination event was detected for each locus when using GARD and one to three events were detected for *DQB* and *DQA*, respectively, using the RDP statistics (Tables S1 and S2). The estimates of population mutation (θ) and recombination (ρ) obtained with the LDhat approach showed that each of these processes has played a significant role in generating the diversity seen among the currently studied MHC beta genes (Table 4). To find out the relative contribution of recombination and point mutations in the evolution of these two currently studied genes in hares, we examined the ratio of average mutation (θ) and recombination rate (ρ). By this measure, mutation was of approximately hundred times more important in the evolution of *DQA* than recombination. In contrast, the recombination rate was about one and a half times greater than mutation in the evolution of the *DQB* gene (Table 4).

**Table 4:** Intralocus recombination in the Tunisian hare sequences measured as r^2^, D’ and G4, and LDhat population mutation (Wattersons θ = 4 Nμ) and population recombination (ρ = 4 Nr).

	r^2^	|D’|	G4	ρ	θ	ρ/θ
*DQA*	0.432 (−0.0071)	0.798 (0.0388)	0.712 (0.0232)	0.126	12.719	0.010
*DQB*	0.001 (−0.1639)	0.194 (−0.0370)	0.191 (−0.0407)	16.771	11.53	1.455

The codons found to be positively selected by all methods are shown in Figs 1 and 2. Five codons (8, 10, 16, 55 and 69) were considered to be under episodic diversifying selection for *DQA* as evidenced by more than one of the applied tests in DATAMONKEY. Six (8, 10, 50, 53, 55 and 76) and three (47, 55 and 69) codons were reported under positive selection for the same locus by PAML (see Table S3) and OMEGAMAP, respectively (Figs 1 and 2). Only site 55 was confirmed by all methods. Twelve codons were suggested to be under negative purifying selection in *DQA* as indicated by the different DATAMONKEY tests.

For *DQB*, five positions (amino acid sites 8, 26, 48, 60, 67) were under episodic diversifying selection in DATAMONKEY. Ten and eleven codons were detected with PAML (codons 13, 14, 26, 32, 37, 48, 57, 59, 60 and 67) and OMEGAMAP (13, 14, 26, 30, 37, 48, 57, 59, 60, 66 and 67), respectively. Four codons (26, 48, 60 and 67) were confirmed by all three methods to be under positive selection. Negative selection in the *DQB* locus was detected in five codons (15, 16, 45, 62 and 72) as suggested by DATAMONKEY tests.

Finally, the outlier test of Beaumont and Nichols^35^ indicated departure from expectation under a neutral evolution scenario for both the *DQA* and the *DQB* locus (Fig. 3), with higher values than the microsatellite loci, in accordance with the codon-based signals of positive selection.

### Statistical Models of *DQA* and *DQB* protein occurrence

In three cases we found models (Table 5) with excellent support from the data (i.e. delta AICc > 10, Null-model insubstantial^36^), in two other cases models with strong support (delta AICc > 8) and in two cases models with some support (delta AICc > 4). Two models had a deviance explained of more than 40%, which is notable, because in multinomial models nominal data are compared with quantitative predictions.

**Table 5:** Results of multinomial models for all seven dependent variables.

variables	dqb1gt		dqb3gt		dqb9gt		dqa7gt		dqa8gt		dqa11gt		hezyg	
	RVI	delta dev	RVI	delta dev	RVI	delta dev	RVI	delta dev	RVI	delta dev	RVI	delta dev	RVI	delta dev
lat	0.68	5.7	1	49.7	1	59.7	0.99	12.2	0.53	5.3	1	17.8	0.89	7.8
long	0.76	5.7	0.35	2.2	0.63	2.1	0.27	1.5	0.98	12.2	0.14	1.1	0.11	1.1
alt	0.13	0.5	0.17	0.6	0.24	0.0	0.15	0.2	0.19	2.1	0.14	1.0	0.15	1.7
dqa_fixedfac	0.6	4.8	0.18	1.5	0.4	3.4								
dqb_fixedfac							0.11	0.1	0.12	0.5	0.25	2.1		
delta AICc	5.98		49.58		59.44		8.41		8.99		16.29		4.4	
dev explained	11.52%		41.96%		40.47%		8.46%		7.71%		15.84%		4.21%	

Except for dqb1gt, always one of the geographic variables had a high RVI and this was multiple times stronger than dqa/dqb (not valid for the heterozygosity models).

In all models except for the “dqb1gt model” geographic variables (latitude or longitude) had a high RVI-value (> = 0.89) indicating a high probability for these variables being in the best model. Furthermore, the delta deviance value of one of the geographic variables was more than ten times higher than the respective co-occurring *DQA* or *DQB* protein variants combinations in four models and eight times higher in the “dqa11gt”-model (Table 5; see also Supplementary Table S4 for estimated coefficients, standard errors, and upper and lower bounds of the 95% confidence interval for all models). This indicates strong support for an effect of the geographic variables on the dependent variables in comparison to the respective co-occurring *DQA* or *DQB* protein variants combinations. In the “dqb1gt-model”, again the performance was very low. Overall, these results suggest a strong effect of geographic variables on the co-occurring *DQA* or *DQB* protein variants. In particular, the proteins of Lecp-DQA*07 and Lecp-DQA*08 did occur more often in homozygous state in Northern Tunisia, with lower mean annual temperatures and higher levels of precipitation, whereas proteins of Lecp DQA*11 were more common in South Tunisia, with higher mean annual temperatures and lower precipitation. For the *DQB* locus, proteins of *Lecp*-DQB*01 were more frequent, in homozygous state, in eastern parts of Tunisia, proteins of *Lecp*-DQB*01 in heterozygous state as well as proteins of *Lecp*-DQB*09 in homozygous and heterozygous state were more common in North Tunisia, whereas proteins of *Lecp*-DQB*03 in homozygous and heterozygous state were more frequent in South Tunisia. Moreover, proteins were generally more common, in heterozygous state, in North Tunisia.

## Discussion

We assessed the MHC diversity and its distribution at two MHC class II genes, *DQA* and *DQB*, in hare populations (*L. capensis*) along a steep ecological gradient in North Africa. We found 17 *DQA* and 26 *DQB* alleles among 142 *L. capensis* individuals. We have analysed the molecular mechanisms of sequence evolution in both loci, i.e., substitution, recombination, and positive selection. We have also described the genetic differentiation and population structure and compared this across both MHC loci and populations. Apart from significant positive selection and recombination events, we found a significant decrease of allelic richness at the two MHC loci from the northern population under Mediterranean humid climate to the southern population in the Sahara region. In addition, we have found a high population substructuring at the two MHC loci compared to supposedly neutral microsatellite markers.

Our results show a high level of polymorphism for the *DQA* and *DQB* loci. Such wide allelic diversity in both allele numbers and pairwise nucleotide distances is characteristic for MHC genes^37^. Moreover, this is in accordance with the results of our previous analyses of the same individuals using mtDNA control region sequences^38^, partial transferrin sequences^39^, allozyme^40^, and microsatellites^38^, which confirms the generally high genetic variability of Tunisian hare populations. Notably, the detected levels of polymorphism of *DQA* and *DQB* were higher than those reported for brown hares (*L. europaeus*) from Austria and Belgium^8,41–43^. Klein^44^ and Takahata *et al*.^45^ suggested that such differences in polymorphism could be due to either a difference in the speed of allelic diversification or in time of their persistence in the gene pool. Both loci in our study have exhibited very similar substitution rates, but a greater recombination rate was recorded for DQB. This effect may be due to a longer persistence of *DQA* diversity in the populations, whereas *DQB* allele diversification may be more likely due to higher intra-locus recombination. Indeed, we found evidence for multiple (intra-allelic) recombination events creating novel *DQA and DQB* haplotypes. According to the different methods used (that vary in their conservative nature), breakpoints at position 120 and 221 were detected by only one test (Tables S1 and S2). For *DQA*, multiple breakpoint positions were obtained and confirmed by more than one of the methods. In contrast, the overall recombination rate (ρ = 16.771) of *DQB* was one and a half times more than the mean mutation rate (θ = 11.53) indicating that the generation of *DQB* diversity is driven by an accumulation of new recombinants relative to new mutations (ρ/θ = 1.455), which is similar to results obtained in other *Lepus* species^43^. For DQA, recombination seems to have a minor effect on its evolution compared to mutations (the ratio ρ/θ = 0.010). In contrast, Wegner^46^ showed that recombination rate and intensity of positive selection were positively correlated in MHC class I and class II sequences of nine different species of fish, suggesting that recombination and gene conversion played a significant role in the evolution of the studied MHC genes.

Genetic differentiation between the studied Tunisian hare populations was high in both MHC class II genes as suggested by high and significant F_ST_ and Dest values. But the same populations showed little genetic structure in the microsatellite loci, which is in accordance with mtDNA and nuclear sequences^39,47^, as well as microsatellite and allozyme genotypes^38,40^ studied earlier in these populations. Therefore, population genetic differentiation at MHC loci could result from either a divergence in qualitative and quantitative parasite pressure that would select for different types of alleles in the different ecoregions but may also be due to differences in modes of mutation and mutation rates between the two marker types. However, high gene flow was concordantly indicated by several markers^38–40^ (microsatellites, allozymes, mtDNA d-loop and transferrin sequences) characterized by different modes of mutation and mutation rates. Therefore, the observed pattern of differentiation only at MHC loci is likely the result of differential selection pressures across the studied populations which is consistent with theoretical expectations^28^.

Indeed, the few frequent alleles (those with allele frequency >10%; Lecp-DQA*07 and Lecp-DQB*09 for NT, Lecp-DQA*08 & Lecp-DQB*01 for CT, Lecp-DQA*11 and Lecp-DQB*03 for ST) vary greatly in each population for each locus. Those predominant alleles were suggested by the PCA to be responsible for the genetic differentiation observed at MHC loci between the three ecoregions. Such differences in allele frequencies between populations have been demonstrated to be mediated by pathogens in affected and unaffected chamois populations^48^. In contrast, cohorts of brown hares characterized by similar quantitative and qualitative pathogens have exhibited the same frequent allele as a sign of stabilizing selection^41^. Moreover, estimations of effective population size and bottleneck signature indicate that the reduced allelic richness of the ST population may be the result of a smaller and/or drifting population. Therefore, the lower MHC polymorphism, in terms of number of alleles, in the ST population may be due to demographic processes, independently from specific parasite pressure. The results indicate that the population structuring at MHC loci is linked to qualitative MHC polymorphism (allelic diversity: different alleles are frequent in different populations) likely shaped by variation in parasite pressure, but the quantitative MHC polymorphism (allelic richness: fewer alleles from North to South) may still be the results of demographic processes.

Both loci showed excess of non-synonymous to synonymous substitutions, a signal of positive selection^49^ that was stronger at the *DQB* locus than for DQA as indicated by a significantly higher number of positively selected sites. In contrast, negative purifying selection was stronger at the *DQA* than in *DQB* locus. In comparison with other studies of lagomorph species, Goüy de Bellocq *et al*.^42^ identified only one codon under positive selection (amino acid 56 in the current study) in thirteen *DQA* alleles of *L. europaeus* populations from Austria and Belgium and four codons (three were identified presently as well: 10, 56, and 70) among 19 *Oryctolagus cuniculus* alleles. For *DQB*, Smith *et al*.^43^ identified two codons (codons 26 and 60) under positive selection among eleven *DQB* alleles in *L. europaeus*, which are also under positive selection in the current study.

**Figure 4:**
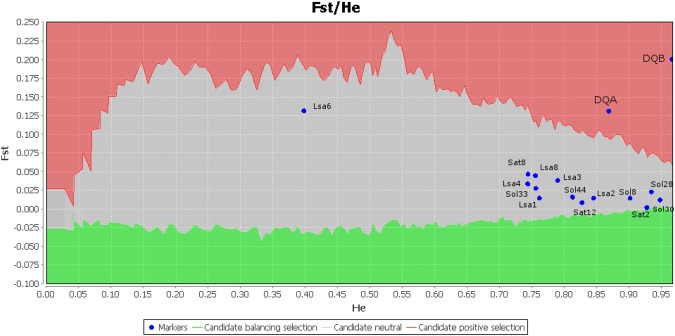
Estimated F_ST_ values from the 16 loci studied (14 microsatellites, *DQA* and *DQB*) plotted as a function of heterozygosity (He) using Lositan software. Gray shading indicates the area on the graph within the confidence limits. *DQA* and *DQB* were found to be under positive selection.

The observed heterozygosity values of MHC loci were rather low in all three regional populations showing a significant deficit. The low heterozygosity might fit to a pattern of recent expansion, as suggested by mitochondrial and nuclear sequences^38,39^ or be due to allelic dropout as different alleles might have similar conformation when using CE-SSCP techniques^1,50^. However, the distribution of MHC heterozygosity suggests that heterozygote advantage is unlikely given the observed positive selection in the studied populations. Indeed, the MHC F_ST_ values were clearly higher than those for microsatellite loci, suggesting an apparent effect of diversifying positive selection. This conclusion was also supported by the outliers test, where the *DQA* and *DQB* loci showed significantly higher mean F_ST_ than predicted from its mean heterozygosity (Fig. 4). Further support for our interpretation comes from our observation of regionally differentiated populations that also suggest diversifying selection maintained by changes of pathogen composition across geographic regions. However, other mechanisms, such as sexual selection and mating system, might drive the evolution of MHC genes^28^. Nevertheless, it is probable that only the effects of pathogens vary both over time and among populations^8^. Host-parasite interactions were suggested to be shaped by environmental conditions (i.e., temperature, nutrient availability; see Brunner and Eizaguirre^17^ for an overview) which can influence parasite species richness, as well as intensity of infestation of hosts. According to Klein and Horejsi^51^ an indirect assessment of parasite infestation can be seen via analysis of MHC class II molecules diversity in the host species, as these molecules primarily correspond to extracellular pathogens, such as bacteria, nematodes, cestodes. Indeed, associations between MHC variants and parasite load have been demonstrated in numerous cases^28^. Here we found significant effects of mean annual temperature and precipitation in the distribution of some MHC proteins/alleles which might indicate regional and climate effects. Concordantly, genetic differentiation and estimation of gene flow indicated regional distribution patterns for MHC loci compared to high genetic admixture in the neutral markers (i.e. microsatellites). Both results suggest that the MHC polymorphism across sampling populations might reflect an adaptation to variation in parasite pressure across the ecological gradient. We expect quantitative and qualitative changes of pathogens from the Mediterranean humid region in the north to the arid Sahara in the south. Indeed, Zvinorova *et al*.^52^ showed that the highest helminths and *Eimeria* infections in goats were observed in the wet season, whereas Pandey *et al*.^53^ suggested direct relationship between rainfall and intensity of infection with gastrointestinal nematodes in goats. Notably, diversity of the mammalian fauna that potentially constitutes a complex pathogen reservoir shows a reduction along the currently studied ecological gradient in Tunisia^54^, probably due to habitat changes and nutrient availability. Moreover, population density of hares from Tunisia seems to decrease from North to South Tunisia (personal observation during hunting trips). This may point towards a combined effect of various aspects, mainly driven by environmental conditions, resulting in the reduction of pathogens in the Sahara region and hence resulting in lower MHC diversity.

Estimation of effective population size showed that Ne estimates were not consistent across methods. This is expected as the accuracy and precision of the different methods to estimate Ne are still uncertain^55^. We followed Wang^56^ who showed that linkage disequilibrium (LD) methods perform dramatically better than the other methods. Ne estimates using this method indicated a similar effective population sizes in all three populations. Moreover, our current Ne estimate for the ST population was less than 400 which was suggested to be the minimum effective population size necessary to maintain genetic variation in the long term in a population^57^. This later population was suggested also by the bottleneck test on neutral microsatellite loci to be under genetic drift. Furthermore, Olivier et Piertney^58^ have demonstrated that selection counters drift to maintain polymorphism at a major histocompatibility complex (MHC) locus in water vole bottlenecked population from Coiresa Island. Therefore, we consider that demographic factors (e.g. bottlenecks or drift) were strong enough to shape the allelic richness in ST population and/or selective pressures were weak to counter the effect of genetic drift.

In conclusion, our present findings from hares along a steep ecological gradient in North Africa indicated that MHC polymorphism and differentiation were shaped by a combination of neutral demographic processes and climate-driven positive selection of MHC genes across relatively short geographical distances. How changes in pathogenic landscape diversity on the one hand, and how immunologic resistance or tolerance of infected hosts on the other hand may interact with local or regional climatic parameters in the evolution of MHC diversity and in the course of adaptation to environmental characteristics needs, however, further studies with particular inclusion of infection patterns of the hosts.

## Methods

### Samples

**Figure 5:**
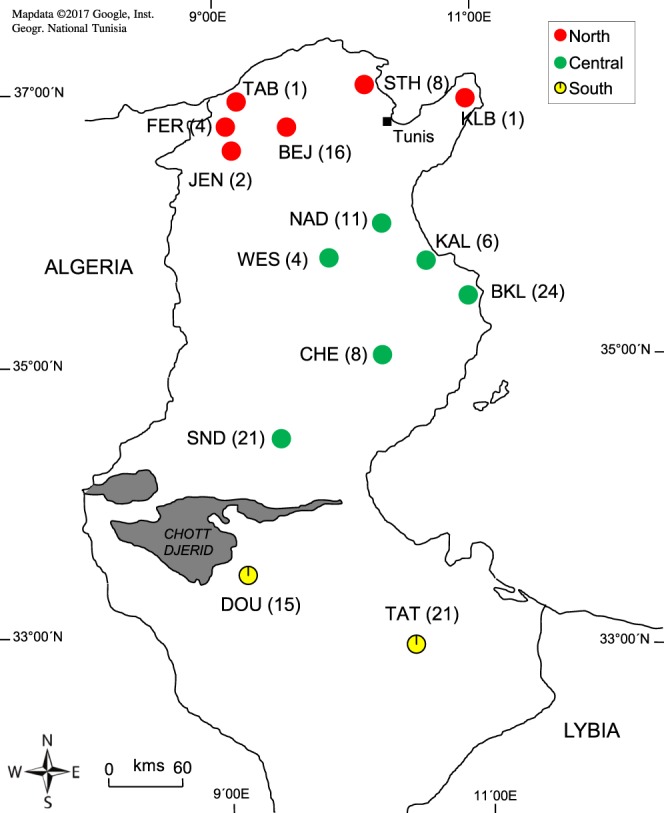
Sampling regions of hares from North, Central and South Tunisia [The original map was created by using Google Maps, https://www.google.tn/maps/@34.6113892,8.7590835,6z?hl=fr and the map of Tunisia was drawn by using POWERPOINT 2007 part of the Microsoft Office Package (https://www.microsoft.com/fr-fr/software-download/office)]. Sample sizes appear in parentheses. Hares were grouped into three populations according to climatic, geographic and phenotypic data.

A total of 142 samples of cape hares (*Lepus capensis*, e.g.^59–61^) were collected during regulars hunts from various biogeographic zones within the major ecoregions in Tunisia. We never manipulated live animals; we got only hare samples during regular hunting season from hunters. All samples were collected with the agreement of the general forest department of Tunisia (authorization no. 2629DGF/DCF/CPN; Ministry of Agriculture, Water Resources and Fishing, Tunisia). Hares were grouped into three populations corresponding to the three main climatic zones in Tunisia (e.g. You *et al*.^62^). Acronyms of sampling localities and positions within northern (NT), central (CT), and southern (ST) Tunisia, as well as sample sizes are given in Fig. 5. The whole sampling area expanded from the Mediterranean humid and sub-humid bioclimatic zones in the north (NT), characterized by cork oak (*Quercus suber*) Mediterranean forest and scrubland (maquis: rosemary, carob tree, mastic tree, thyme, lavender), across the semi-arid central part of Tunisia (CT) to the arid and hyper-arid Sahara in the south (ST), with typical plants that have adapted to dry conditions such as *Acacia tortilis* subsp. *raddiana*, *Vachellia* spp., *Stipagrostis pungens* vegetation^62,63^. Our study area represents a remarkably steep climatic and ecological gradient across a straight distance of less than 500 km, with means of annual precipitation and temperature ranging between 916 mm and 16.3 °C in the north and 77 mm and 21.26 °C in the south.

### PCR amplification, CE-SSCP and Sequencing

Total genomic DNA was extracted using the GenElute Mammalian Genomic DNA Miniprep kit (Sigma-Aldrich) from diverse tissues (liver, skeletal muscles, ear cartilage, tongue) of each specimen^5,39,47,60^.

Amplification of exon 2 fragments for two MHC class II genes (*DQA* and *DQB*) were performed according to Goüy de Bellocq *et al*.^42^ and Smith *et al*.^43^. The amplified 258 bp and 249 bp (including primers) correspond to the total exon 2 of DQA and DQB genes, respectively. We used capillary electrophoresis single-strand conformation polymorphism (CE-SSCP) to screen allelic diversity in the above mentioned genes. Amplification was carried out using the Multiplex PCR kit (Qiagen) following the manufacturer’s instructions in a final reaction volume of 10 μl. Forward primers for both gene segments were 6’FAM-labelled and reverse primers were NED-labelled.

We selected individuals with diverse SSCP patterns, representing all identified alleles, to investigate sequence variation at both *DQA* and *DQB* loci, respectively. The genes were amplified using the same procedure as in Goüy de Bellocq *et al*.^42^ and Smith *et al*.^43^, but using non-labelled primers. As homozygous states were detected by SSCP analysis for all alleles in both gene segments, we directly sequenced the PCR product of the corresponding samples. Among all currently analysed samples, only eight were not screened with SCCP. These eight samples were directly sequenced using the same primers and the program phase^64^ was used to separate alleles in heterozygous samples.

For comparison between MHC and neutral genetic variation, genotype data of 14 microsatellite loci of 134 individuals out of the specimens analysed in the current study were used from a previous study^38^.

### Data analysis

#### Population genetic parameters

Sequences were edited and aligned by eye using the BioEdit v.7.2.5 program©, 1997-2013^65^. Allele frequencies, mean number of alleles (A), observed (Ho) and expected (He) heterozygosity were calculated for each locus and for each regional population (NT, CT, ST) with GENETIX^66^. Tests of deviation from Hardy–Weinberg equilibrium and linkage disequilibrium were calculated using GENEPOP 4.0^67^ and GENETIX^66^, respectively. The allelic numbers for the cape hare sequences were assigned according to the guidelines of Klein *et al*.^68^ and begin at Lecp-DQA*04 and Lecp-DQB*01 for DQA and DQB, respectively. The FSTAT vers. 2.9.3.2. program^69^ was used to calculate locus-specific values of allelic richness (Rs) based on a rarefaction approach to account for different sample sizes. Allelic richness calculation was based on minimum sample size of 29 and 18 individuals for MHC and microsatellite loci, respectively.

Differentiation between regional populations was determined by calculation of standardized pairwise F_ST_ (10 000 permutations) in GENETIX^66^ and also Jost’s D (Dest)^70^ as a more robust measure than F_ST_ using GenAlEx 6.5^71^. In addition, an analysis of molecular variance (AMOVA) from the MHC data was calculated for the three eco-regions (NT, CT, ST) using the Arlequin 3.5 program^72^. The same genetic parameters were also estimated for genotypes at 14 microsatellite loci obtained earlier for the same individuals^38^ for NT, CT, ST.

Furthermore, in order to identify the contribution of the different MHC alleles to the population structure, a principal components analysis (PCA) was performed in ADE-4^73^, using the genotype data at both MHCII loci. Coordinates obtained for each allele were used for scatter plots using Past software^74^.

Finally, as demographic events might have affected the observed pattern of diversity and differentiation in MHC loci, we first use the “Wilcoxon signed-ranks test”^75^ implemented in the bottleneck software to test for bottleneck in the microsatellite data. As recommended by Piry*et al*.^75^, we used the Two Phase Mutation model (TPM) and the Stepwise Mutation Model (SMM).We then calculated effective population size using the neutral microsatellite data using Ne estimator^76^. Three different methods based on the information of linkage disequilibrium (LD), heterozygote excess (HE) and molecular coancestry (MC) were used.

#### Recombination analysis

The assumptions of several methods of sequence analysis are violated if recombination is present^77^. To check for the presence of recombination in the alignment of our sequences, we first used PERMUTE (included in the software package OMEGAMAP^78^), which computes three different statistics (r^2^, D, and G4) based on the correlation between physical distance of sites and their linkage disequilibrium. The RDP, GENECONV, MAXCHI, CHIMAERA and 3SEQ implemented in the RDP3 package^79^ were all employed to detect recombination breakpoint locations. In the latter analyses we used a significance level of 0.05. We also calculated the population scaled recombination rate ρ and mutation rate θ by using LDhat recombination rate scan implemented in theRDP3 package^79^. Finally, the GARD method^80^ was employed to search for possible recombination partitions.

#### Detecting positive selection

In order to infer evidence of positive selection, three different methods that compare the rates of synonymous and non-synonymous substitutions separately for every single codon were used. We chose to use HYPHY^81^ as implemented in the DATAMONKEY webserver (http://www.datamonkey.org/83; last accessed 30^th^ January 2017), OMEGAMAP^78^ and CODEML (PAML 4 package^82^). The first two software packages have the important advantage of taking recombination into account whereas CODEML does not account for that.

The OmegaMap program is based on a Bayesian population genetics approximation to the coalescent theory and generates means and credible intervals for the selection parameter (dN/dS = ω) and recombination rate (ρ = 4Nr) for each codon (N and r represent the effective population size and the per codon rate of recombination, respectively). We used the same parameters as in Smith *et al*.^43^. Two Markov chain Monte Carlo runs of 250 000 iterations (25 000 iteration burn-in) for *DQA* and 400 000 iterations (40 000 iteration burn-in) for *DQB* on population allele frequencies at each locus, were compared for convergence. Codons are considered as positively selected with posterior probabilities greater than 95%.

In DATAMONKEY, we first identified recombination break points with genetic algorithm recombination detection (GARD^80^) that infers phylogenies for each putative non recombinant fragment. The output was used to run four different maximum likelihood methods for detection of selection: SLAC (single likelihood ancestral counting), FEL (fixed effects likelihood), REL (random effects likelihood), and Mixed Effects Model of Evolution (MEME)^83^. Significance levels of P < 0.25 in SLAC and FEL and P < 0.05 in MEME and Bayes factors >50 in REL were considered as indicating positively selected sites. We considered a codon to be positively selected only if it was identified by at least two of the methods implemented^77,84^.

Several studies^42,85,86^ that used CODEML to test for site specific positive selection in MHC data have shown its robustness although it does not account for recombination events. Two pairs of models were compared using this program: M1a (neutral model) was compared to M2a (adaptive model) and M7 (beta) was compared to M8 (beta plus omega). Pairwise comparisons on nested models were performed using the likelihood ratio test (LRT): twice the log-likelihood difference was compared with a χ^y^distribution with degrees of freedom equal to the difference in the number of parameters between both models. When the LRT was significant, a Bayes Empirical Bayes (BEB) method was used to calculate the posterior probabilities of codon classes in models M2a and M8. Posterior probabilities of >0.95 were considered as supportive under the BEB method^87^. We considered a codon to be positively selected if it was identified by M2a or M8 or both.

In addition to the applied tests to infer molecular signature of positive selection, we used the model-based approach of Beaumont and Nichols^35^, based on MHC genotypes, implemented in Lositan^88^ to compare the observed F_ST_ values estimated at each locus to a null distribution of F_ST_ conditional on heterozygosity. *DQA*, *DQB* and fourteen microsatellite loci^38^ were tested for neutrality under 50000 simulations, estimated neutral mean F_ST_, infinite alleles mutation model, 99% confidence interval and false discovery rate of 0.1%.

#### Testing for variation of genetic diversity between regions and for effects of climate variables on MHC alleles and heterozygosity

First, we used locus specific allelic richness of MHC and microsatellite loci in the three regional populations to run a linear mixed effects model (lmer) to check for significant variation of allelic richness from North to South Tunisia. Under a positive selection scenario for MHC loci we expected that allelic richness at both MHC loci varies between sampling regions in the case of different pathogen pressure. However, genetic diversity (allelic richness) for selectively neutral markers like microsatellites was not expected to vary between populations, as population genetic results indicated high gene flow across the whole study region^38–40^. Thus, with a hypothesized higher pathogenic diversity in the more climatically diverse NT population, MHC diversity at the two studied loci was expected to exceed the neutral microsatellite diversity particularly in NT. In the model we used locus specific allelic richness as obtained by FSTAT as dependent variable, regional population (NT, CT, ST) and locus type (MHC or microsatellite) as fixed factors and locus as random variable to account for potential locus-specific effects (particularly among the microsatellites) and specifically tested for a significant locus type by population interaction effect. The model syntax was as follows: lmer (random effect: ~ 1I locus; locus specific allelic richness ~ population * locus type). We also tested if allelic richness varies across the ecological gradient independently from microsatellite diversity using a general linear model of the syntax: MHC allelic richness ~ region; where MHC allelic richness means locus-specific allelic richness (for DQA and DQB) and region means NT or (CT & ST). CT and ST populations were combined because allelic richness values (see Table 1) were higher in NT but similarly lower in CT and ST.

Second, we used the statistical software package R 2.15.0 (R Development Core Team, 2011) to run multinomial log-linear models separately for the most frequent protein variants at the *DQA* (07, 08, 11) and the *DQB* (01, 03, 09) loci as response variables and geographical latitude, longitude, altitude as well as the protein combination at the respective co-occurring MHC locus as independent variables. However, mean annual temperature (r = −0.796) and annual precipitation (r = 0.831) correlated too closely with latitude to add them in the model, and latitude has the clearest effect of these three variables on the response variable (see Supplementary Table S5). Thus, hereafter latitude will be interpreted as surrogate for climate, acknowledging that other abiotic factors like soil or wetness also might be covered by this variable. Mean annual temperature, annual precipitation and altitude were obtained from WORLDCLIM data set for 2.5 min intervals (Version 1.4, http://www.worldclim.org/bioclim.htm) and were automatically extracted using DIVA-GIS ver. 7.5. We also run a multinomial log-linear model with individual heterozygosity in *DQA* and *DQB* loci as response variable and the same geographic variables as independent variables, expecting a higher level in NT (in parallel to allelic richness).

The model syntaxes were as follows (using the package nnet):

1. Multinom (*DQA* protein class ~ latitude + longitude + altitude + as.factor (co-occurring *DQB* protein class)),
  where the two co-occurring protein classes represented either any *DQB* protein combination of the most prevalent proteins *01, 03, 09*, on the one hand and any other protein combination on the other,
2. Multinom (*DQB* protein class ~ latitude * longitude + altitude + as.factor (co-occurring *DQA* protein class)),
  where the two co-occurring protein classes represented either any *DQA* protein combination of the most prevalent proteins 07, 08, 11 on the one hand and any other protein combination on the other,
3. Multinom (MHC heterozygosity ~ latitude * longitude + altitude).

We used an information-theoretic approach and techniques of model selection and multi-model inference^36^ (i.e., model ranking and model averaging) to obtain the best model explaining the data. Based on the global models (see syntaxes above), we run all possible models including the respective null-models and calculated the probability of each possible model being the best model using the Akaike weight based on AICc values (i.e., corrected for small sample sizes). Adding up the weight of all models in which a given variable is present gives the “Relative Variable Importance”, RVI, Table 5). RVI is an estimate of the absolute probability for a variable of being in the best model explaining this particular dataset. For each variable we also calculated the so-called delta deviance, i.e. the difference in deviance between the full model and the model after dropping this variable. In the cases of completely uncorrelated variables (which is almost the case here), delta deviance gives a relative estimate on the effect strength of one variable to the other, e.g. the effect of latitude on dqb3gt is 49.7/1.5 ≈ 32 times stronger than the effect of the respective co-occuring dqa genotype. Additionally we report the estimates, standard errors and the upper and lower 95% confidence interval (see Supp2), which indicates the precision of the estimate. We do not use these outputs to interpret the importance of the variables, because this would almost like using p-values in a Null-hypothesis testing framework, which should be avoided in an information-theoretic approach^36^. For each model we report delta AICc (difference in AICc between the best model and the intercept-only model) and the explained deviance as measure of goodness of fit (Table 5).

### Accession codes

Sequence data from this article can be found in the GenBank under the accession numbers: MH346126-MH346168.

## Electronic supplementary material

Table S1 to S5
