# Supplementary material for: Positive selection and climatic effects on MHC class II gene diversity in hares (Lepus capensis) from a steep ecological gradient
Source: Sci Rep. 2018 Jul 31;8:11514. doi: 10.1038/s41598-018-29657-3 (PMC6068193; doi:10.1038/s41598-018-29657-3)
Supplement: Supplementary file 1 — Table S1 to S5 [file 41598_2018_29657_MOESM1_ESM.docx]

Positive selection and climatic effects on *MHC* class II gene diversity in hares (*Lepus capensis*) from a steep ecological gradient

Asma Awadi^1^, Hichem Ben Slimen^1,2^, Steve Smith^3^, Felix Knauer^3^, Mohamed Makni^1^, Franz Suchentrunk^3^

^1^Unité de Recherche Génomique des Insectes Ravageurs des Culturesd’Intérêt Agronomique, Faculty of Sciences of Tunis, University of Tunis El Manar, 2092 Tunis, Tunisia. ^2^Institut Supérieur de Biotechnologie de Béja, University of Jendouba, Avenue Habib Bourguiba Béja 9000, BP. 382, Béja, Tunisia. ^3^Research Institute of Wildlife Ecology, University of Veterinary Medicine Vienna, Savoyenstrasse 1, 1160 Vienna, Austria. Correspondence and requests for materials should be addressed to A.A. (email: awadiasma@gmail.com)

**Table S1. Recombination events detected by various methods in the RDP3 program.** * means the actual breakpoint position is undetermined. NS is short for “not significant”.

| Locus | Recombination Event Number | Breakpoint Positions in alignment | | Recombinant Sequence(s) | Minor Parental Sequence(s) | Major Parental Sequence(s) | Detection methods | | | | |
| --- | --- | --- | --- | --- | --- | --- | --- | --- | --- | --- | --- |
|  |  | Begin | End |  |  |  | RDP | GENECONV | Maxchi | Chimaera | 3Seq |
| DQA | 1 | 167* | 104 | Allele25 | Allele03LE | Unknown (allele17) | NS | NS | <0.01 | <0.05 | <0.05 |
|  |  |  |  |  |  |  |  |  |  |  |  |
|  | 2 | 70* | 154* | Allele 16 | Allele17 | Unknown (allele25) | NS | NS | <0.05 | <0.01 | NS |
|  |  |  |  |  |  |  |  |  |  |  |  |
|  | 3 | 120* | 154 | Allele25 | Unknown (allele16) | Allele03L | NS | NS | NS | NS | <0.05 |
| DQB | 1 | 52 | 198 | Allele17 | Allele34 | Allele31 | NS | NS | NS | NS | <0.05 |

**Table S2. Breakpoints detected by GARD method**

| Breakpoint | LHS p-value | RHS P-value | significance |
| --- | --- | --- | --- |
| DQA97 | 0.0002 | 0.0002 | <0.001 |
| DQB119 | 0.0002 | 0.0002 | <0.001 |

**Table S3** Results of PAML analysis. Model = name of the model; ***ln L*** = the natural logarithm of the likelihood obtained for every model; Parameters = estimates of ω values and proportion of codons that belong to each ω class; Sites: position in the corresponding gene of each site under positive selection; p(*Δ*LRT) = P-values of the log likelihood ratio test for model comparisons, null models are shown in brackets.

.

| ***Model*** | ***ln L*** | | ***DQA*** | | | ***DQB*** | | |
| --- | --- | --- | --- | --- | --- | --- | --- | --- |
|  | ***DQA*** | ***DQB*** | ***Parameters*** | ***Sites^a^*** | ***p (ΔLRT)*** | ***Parameters*** | ***Sites^a^*** | ***p (ΔLRT)*** |
| M0 (one ratio) | -907.8941 | -1778.0623 | ω= 0.670 |  |  | ω= 0.489 |  |  |
| M1a (nearly neutral) | -881.3254 | -1583.4541 | p0= 0.653, p1= 0.347 |  | <0.001  (M0) | p0= 0.680, p1= 0.320 |  | <0.001  (M0) |
| M2a (positive selection) | -870.4970 | -1554.4197 | p0= 0.644, p1= 0.226, p2= 0.130, ω2= 4.249 | **2, 4**, 28, 39, 44, 47**, 49,** 63**, 70** | <0.001  (M1a) | p0= 0.663, p1= 0.194, p2= 0.143, ω2= 3.346 | 11**, 12, 24,** 26, 32**, 35, 46, 55,** 57**, 58, 65** | >0.05  (M1a) |
| M3 (discrete) | -870.7492 | -1553.9194 | p0= 0.431, p1= 0.415, p2= 0.154, ω0=0.000, ω1=0.503, ω2=3.787 |  | <0.001  (M0) | p0= 0.648, p1=0.170, p2= 0.182, ω0=0.006, ω1=0.682, ω2=2.854 |  | <0.001  (M0) |
| M7 (beta) | -881.6118 | -1575.4875 | p= 0.014, q= 0.019 |  |  | p=0.015, q=0.050 |  |  |
| M8 (beta and omega) | -870.9533 | -1554.4205 | p0= 0.854, p1= 0.146, p= 0.291, q= 0.790, ω= 3.900 | **2,** 3**, 4,** 19, 28, 39, 41**, 44, 47, 49,** 63**, 70** | <0.001  (M7) | p0=0.835, p1=0.165, p=0.054, q=0.296, ω=2.815 | **11, 12,** 16**, 24,** 26, 28**, 32, 35,** 36, 43**, 46, 55, 57, 58, 65,** 73 | <0.001  (M7) |

**^a^**: BEB: P(ω>1)>0.50; P(ω>1)>0.95 is shown in bold

**Table S4.** Averaged model estimates of coefficients, standard errors and upper and lower bounds of the 95% confidence interval for all models

| **dqb1gt** | **Estimate** | **Std. Error** | **95%-CI_low** | **95%-CI_up** |
| --- | --- | --- | --- | --- |
| present heterozygous((Intercept)) | -2.60E+01 | 2.68E+01 | -7.86E+01 | 2.65E+01 |
| present homozyogous((Intercept)) | -8.49E+00 | 7.90E+00 | -2.40E+01 | 7.00E+00 |
| present heterozygous(lat) | **6.55E-01** | **3.03E-01** | **6.22E-02** | **1.25E+00** |
| present homozyogous(lat) | -8.49E-02 | 2.36E-01 | -5.47E-01 | 3.78E-01 |
| present heterozygous(long) | 5.32E-01 | 4.35E-01 | -3.21E-01 | 1.38E+00 |
| present homozyogous(long) | **1.06E+00** | **4.66E-01** | **1.48E-01** | **1.97E+00** |
| present heterozygous(alt) | 2.39E-04 | 2.05E-03 | -3.78E-03 | 4.26E-03 |
| present homozyogous(alt) | 5.42E-04 | 2.35E-03 | -4.06E-03 | 5.14E-03 |
| present heterozygous(dqa_fixedfac) | 7.30E+00 | 2.88E+01 | -4.92E+01 | 6.38E+01 |
| present homozyogous(dqa_fixedfac) | 1.00E+00 | 1.10E+00 | -1.16E+00 | 3.16E+00 |
|  |  |  |  |  |
| **dqb3gt** |  |  |  |  |
| present heterozygous((Intercept)) | 56.172689 | 13.502579 | 2.97E+01 | 8.26E+01 |
| present homozygous((Intercept)) | 67.379041 | 22.080507 | 2.41E+01 | 1.11E+02 |
| present heterozygous(lat) | **-1.665275** | **0.389547** | **-2.43E+00** | **-9.02E-01** |
| present homozygous(lat) | **-1.903519** | **0.570492** | **-3.02E+00** | **-7.85E-01** |
| present heterozygous(long) | -0.406337 | 0.671593 | -1.72E+00 | 9.10E-01 |
| present homozygous(long) | -1.455437 | 0.950319 | -3.32E+00 | 4.07E-01 |
| present heterozygous(alt) | -0.001468 | 0.002443 | -6.26E-03 | 3.32E-03 |
| present homozygous(alt) | -0.001995 | 0.004695 | -1.12E-02 | 7.21E-03 |
| present heterozygous(dqa_fixedfac) | 1.075335 | 0.993666 | -8.72E-01 | 3.02E+00 |
| present homozygous(dqa_fixedfac) | 0.124286 | 0.963508 | -1.76E+00 | 2.01E+00 |
|  |  |  |  |  |
| **dqa7gt** |  |  |  |  |
| present heterozygous((Intercept)) | -8.86E+00 | 5.65E+00 | -1.99E+01 | 2.20E+00 |
| present homozygous((Intercept)) | -6.54E+01 | 2.95E+01 | -1.23E+02 | -7.57E+00 |
| present heterozygous(lat) | 2.58E-01 | 1.53E-01 | -4.12E-02 | 5.57E-01 |
| present homozygous(lat) | **1.77E+00** | **7.68E-01** | **2.66E-01** | **3.28E+00** |
| present heterozygous(long) | -4.38E-01 | 2.97E-01 | -1.02E+00 | 1.45E-01 |
| present homozygous(long) | -3.22E-02 | 6.69E-01 | -1.34E+00 | 1.28E+00 |
| present heterozygous(alt) | 8.05E-04 | 1.16E-03 | -1.46E-03 | 3.07E-03 |
| present homozygous(alt) | -9.28E-04 | 2.56E-03 | -5.95E-03 | 4.10E-03 |
| present heterozygous(dqb_fixedfac) | 1.40E-01 | 4.29E-01 | -7.01E-01 | 9.81E-01 |
| present homozygous(dqb_fixedfac) | -1.01E-01 | 8.97E-01 | -1.86E+00 | 1.66E+00 |
|  |  |  |  |  |
| **dqa8gt** |  |  |  |  |
| present heterozygous((Intercept)) | -8.86E+00 | 5.65E+00 | -1.99E+01 | 2.20E+00 |
| present homozygous((Intercept)) | -6.54E+01 | 2.95E+01 | -1.23E+02 | -7.57E+00 |
| present heterozygous(lat) | 2.58E-01 | 1.53E-01 | -4.12E-02 | 5.57E-01 |
| present homozygous(lat) | **1.77E+00** | **7.68E-01** | **2.66E-01** | **3.28E+00** |
| present heterozygous(long) | -4.38E-01 | 2.97E-01 | -1.02E+00 | 1.45E-01 |
| present homozygous(long) | -3.22E-02 | 6.69E-01 | -1.34E+00 | 1.28E+00 |
| present heterozygous(alt) | 8.05E-04 | 1.16E-03 | -1.46E-03 | 3.07E-03 |
| present homozygous(alt) | -9.28E-04 | 2.56E-03 | -5.95E-03 | 4.10E-03 |
| present heterozygous(dqb_fixedfac) | 1.40E-01 | 4.29E-01 | -7.01E-01 | 9.81E-01 |
| present homozygous(dqb_fixedfac) | -1.01E-01 | 8.97E-01 | -1.86E+00 | 1.66E+00 |
|  |  |  |  |  |
| **dqb9gt** |  |  |  |  |
| present heterozygous((Intercept)) | -84.420905 | 26.71964 | -1.37E+02 | -3.21E+01 |
| present homozygous((Intercept)) | -64.346285 | 21.381092 | -1.06E+02 | -2.24E+01 |
| present heterozygous(lat) | **2.512322** | **0.63243** | **1.27E+00** | **3.75E+00** |
| present homozygous(lat) | **1.939417** | **0.511297** | **9.37E-01** | **2.94E+00** |
| present heterozygous(long) | -1.12179 | 0.596062 | -2.29E+00 | 4.65E-02 |
| present homozygous(long) | -1.16129 | 0.600127 | -2.34E+00 | 1.50E-02 |
| present heterozygous(alt) | 0.001798 | 0.002275 | -2.66E-03 | 6.26E-03 |
| present homozygous(alt) | 0.00241 | 0.002205 | -1.91E-03 | 6.73E-03 |
| present heterozygous(dqa_fixedfac) | -1.556394 | 1.08408 | -3.68E+00 | 5.68E-01 |
| present homozygous(dqa_fixedfac) | 0.286903 | 1.438039 | -2.53E+00 | 3.11E+00 |
|  |  |  |  |  |
| **dqa11gt** |  |  |  |  |
| present heterozygous((Intercept)) | 28.9856916 | 8.7675808 | 1.18E+01 | 4.62E+01 |
| present homozygous((Intercept)) | 21.389111 | 7.5251679 | 6.64E+00 | 3.61E+01 |
| present heterozygous(lat) | **-0.883582** | **0.2541073** | **-1.38E+00** | **-3.86E-01** |
| present homozygous(lat) | **-0.6671066** | **0.2157816** | **-1.09E+00** | **-2.44E-01** |
| present heterozygous(long) | -0.4229702 | 0.5332481 | -1.47E+00 | 6.22E-01 |
| present homozygous(long) | -0.2384119 | 0.4683614 | -1.16E+00 | 6.80E-01 |
| present heterozygous(alt) | -0.0007643 | 0.0019553 | -4.60E-03 | 3.07E-03 |
| present homozygous(alt) | -0.0015078 | 0.001731 | -4.90E-03 | 1.88E-03 |
| present heterozygous(dqb_fixedfac) | -0.2351349 | 0.6974966 | -1.60E+00 | 1.13E+00 |
| present homozygous(dqb_fixedfac) | 0.930204 | 0.7125741 | -4.66E-01 | 2.33E+00 |
|  |  |  |  |  |
| **hezyg** |  |  |  |  |
| one heterozygous((Intercept)) | 20.28815 | 10.098627 | 4.95E-01 | 4.01E+01 |
| both homozygous((Intercept)) | 15.600848 | 9.517171 | -3.05E+00 | 3.43E+01 |
| one heterozygous(lat) | **-0.612923** | **0.218928** | **-1.04E+00** | **-1.84E-01** |
| both homozygous(lat) | **-0.482914** | **0.229617** | **-9.33E-01** | **-3.29E-02** |
| one heterozygous(long) | 0.068315 | 0.37266 | -6.62E-01 | 7.99E-01 |
| both homozygous(long) | 0.168365 | 0.40587 | -6.27E-01 | 9.64E-01 |
| one heterozygous(alt) | 0.001165 | 0.001451 | -1.68E-03 | 4.01E-03 |
| both homozygous(alt) | 0.001421 | 0.001555 | -1.63E-03 | 4.47E-03 |

**Table S5.** Choice of correlated geographic/climate variables. Latitude (lat) was best ranked in two of three models and second best in the third model.

| **model** |  |  |  | **dropped variable** | **AICc** | **rank** |
| --- | --- | --- | --- | --- | --- | --- |
| cbind(dqa7gt,dqa8gt,dqa11gt)~long+alt+bio1+bio12+dqb_fixedfac |  |  |  | lat | 1191.877 | 1 |
| cbind(dqa7gt,dqa8gt,dqa11gt)~lat+long+alt+bio12+dqb_fixedfac |  |  |  | bio1 | 1192.682 | 2 |
| cbind(dqa7gt,dqa8gt,dqa11gt)~lat+long+alt+bio1+dqb_fixedfac |  |  |  | bio12 | 1193.092 | 3 |
|  |  |  |  |  |  |  |
| cbind(dqb1gt,dqb3gt,dqb9gt)~long+alt+bio1+bio12+dqa_fixedfac |  |  |  | lat | 1056.451 | 2 |
| cbind(dqb1gt,dqb3gt,dqb9gt)~lat+long+alt+bio12+dqa_fixedfac |  |  |  | bio1 | 1056.489 | 3 |
| cbind(dqb1gt,dqb3gt,dqb9gt)~lat+long+alt+bio1+dqa_fixedfac |  |  |  | bio12 | 1054.486 | 1 |
|  |  |  |  |  |  |  |
| hezyg~long+alt+bio1+bio12 |  |  |  | lat | 255.0768 | 1 |
| hezyg~lat+long+alt+bio12 |  |  |  | bio1 | 255.3312 | 2 |
| hezyg~lat+long+alt+bio1 |  |  |  | bio12 | 258.4684 | 3 |
